# Supplementary material for: Identification of NUDT15 gene variants in Amazonian Amerindians and admixed individuals from northern Brazil
Source: PLoS One. 2020 Apr 15;15(4):e0231651. doi: 10.1371/journal.pone.0231651 (PMC7159207; doi:10.1371/journal.pone.0231651)
Supplement: S1 Table — (PDF) [file pone.0231651.s001.pdf]

Identification of *NUDT15* gene variants in Amazonian Amerindians and admixed individuals from northern Brazil.

Investigating *NUDT15* gene variants in Amerindians and population from northern Brazil

Juliana Carla Gomes Rodrigues[1], Tatiane Piedade de Souza[1], Lucas Favacho Pastana[1], André Maurício Ribeiro dos Santos[2], Marianne Rodrigues Fernandes[1], Pablo Pinto[1,2], Alayde Vieira Wanderley[3], Sandro José de Souza[4], José Eduardo Kroll[4], Adenilson Leão Pereira[2], Leandro Magalhães[2], Laís Reis das Mercês[2], Amanda Ferreira Vidal[2], Tatiana Vinasco-Sandoval[2], Giovanna Chaves Cavalcante[2], João Farias Guerreiro[2], Paulo Pimentel Assumpção[1], Ândrea Ribeiro-dos-Santos[1,2], Sidney Santos[1,2], Ney Pereira Carneiro dos Santos[1,2*].

[1]Núcleo de Pesquisas em Oncologia, Belém, Pará, Brazil.

[2]Laboratório de Genética Humana e Médica, Instituto de Ciências Biológicas, Universidade Federal do Pará, Belém, Pará, Brazil.

[3]Hospital Ophir Loyola, Departamento de Pediatria, Belém, Pará, Brazil

[4]Brain Institute, Universidade Federal do Rio Grande do Norte, Natal, Rio Grande do Norte, Brazil.

Supplementary Table 1. Name, location and number of individuals in each population group studied.

Acronym	Populations	Geographic coordinates*	Number of individuals
AKW	Asurini do Koatinemo	-4.255843, -52.276504	5
ARA	Arara/Arara do Iriri	-3.746185, -53.049108	7
ARW	Araweté	-4.929118, -52.477536	6
AST	Asurini do Trocará	-3.571818, -49.705560	16
AWA	Awa-Guajá	-3.336853, -46.453796	8
CAR	Caripuna	3.559476, -51.491796	1
JUR	Juruna	-3.473968, -51.813613	2
MUN	Munduruku	-6.437803, -57.965514	1
ODJ	Xikrin Odjá	-6.217526, -50.781783	2
PTJ	Zo'é	-0.128339, -55.765829	5
WPI	Wayãpy	1.209187, -52.770094	5
XIK	Xikrin do Cateté	-4.592940, -51.538733	6
BAP	Belém	-1.455830, -48.504440	84
