# Supplementary material for: Identification of NUDT15 gene variants in Amazonian Amerindians and admixed individuals from northern Brazil
Source: PLoS One. 2020 Apr 15;15(4):e0231651. doi: 10.1371/journal.pone.0231651 (PMC7159207; doi:10.1371/journal.pone.0231651)
Supplement: S2 Table — CML = Colombians from Medellin, Colombia; MXL: Mexican Ancestry from Los Angeles USA; PEL = Peruvians from Lima, Peru; PUR = Puerto Ricans from Puerto Rico. (PDF) [file pone.0231651.s002.pdf]

Identification of *NUDT15* gene variants in Amazonian Amerindians and admixed individuals from northern Brazil.

Investigating *NUDT15* gene variants in Amerindians and population from northern Brazil

Juliana Carla Gomes Rodrigues[1], Tatiane Piedade de Souza[1], Lucas Favacho Pastana[1], André Maurício Ribeiro dos Santos[2], Marianne Rodrigues Fernandes[1], Pablo Pinto[1,2], Alayde Vieira Wanderley[3], Sandro José de Souza[4], José Eduardo Kroll[4], Adenilson Leão Pereira[2], Leandro Magalhães[2], Laís Reis das Mercês[2], Amanda Ferreira Vidal[2], Tatiana Vinasco-Sandoval[2], Giovanna Chaves Cavalcante[2], João Farias Guerreiro[2], Paulo Pimentel Assumpção[1], Ândrea Ribeiro-dos-Santos[1,2], Sidney Santos[1,2], Ney Pereira Carneiro dos Santos[1,2*].

[1]Núcleo de Pesquisas em Oncologia, Belém, Pará, Brazil.

[2]Laboratório de Genética Humana e Médica, Instituto de Ciências Biológicas, Universidade Federal do Pará, Belém, Pará, Brazil.

[3]Hospital Ophir Loyola, Departamento de Pediatria, Belém, Pará, Brazil

[4]Brain Institute, Universidade Federal do Rio Grande do Norte, Natal, Rio Grande do Norte, Brazil.

Supplementary Table 2. Allelic frequency independently for each of the Amerindian populations (N ≥ 5), of the admixed Brazilian population, of the subpopulations of the American group from the 1000 genomes, and of the southeast Brazilian samples. CML = Colombians from Medellin, Colombia; MXL: Mexican Ancestry from Los Angeles USA; PEL = Peruvians from Lima, Peru; PUR = Puerto Ricans from Puerto Rico.

Reference SNP Id	Allele Frequencies														
	NAM								BAP	AMR				SUD	
	AKW	ARA	ARW	AST	AWA	PTJ	WPI	XIK	-	CLM	MXL	PEL	PUR	ABraOm	Bpimed
rs1272632214	0.100	0.143	0.083	0.031	0	0.300	0.200	0.100	0.0680	0.021	0.031	0.106	0.005	0.001	0.005
rs147390019	0.200	0.286	0	0.125	0	0	0.200	0.100	0.0067	0.016	0.016	-	-	0.004	-
rs116855232	0.100	0.143	0.083	0.031	0	0.300	0.200	0.100	0.0938	0.021	0.047	0.118	0.005	0.012	-
