# Supplementary material for: Identification of NUDT15 gene variants in Amazonian Amerindians and admixed individuals from northern Brazil
Source: PLoS One. 2020 Apr 15;15(4):e0231651. doi: 10.1371/journal.pone.0231651 (PMC7159207; doi:10.1371/journal.pone.0231651)
Supplement: S1 Dataset — (PDF) [file pone.0231651.s003.pdf]

Anonymised dataset of the Amazonian Amerindian populations

SAMPLE	rs1272632214	rs147390019	rs374594155	rs116855232
NAM1	wt/wt	*NA*	*NA*	wt/wt
NAM2	wt/wt	*NA*	*NA*	wt/wt
NAM3	wt/wt	*NA*	*NA*	wt/wt
NAM4	wt/VAR	*NA*	*NA*	wt/VAR
NAM5	wt/wt	VAR/VAR	*NA*	wt/wt
NAM6	wt/wt	*NA*	wt/wt	wt/wt
NAM7	wt/VAR	*NA*	*NA*	wt/VAR
NAM8	wt/VAR	wt/wt	*NA*	wt/VAR
NAM9	wt/wt	*NA*	*NA*	wt/wt
NAM10	wt/wt	*NA*	*NA*	wt/wt
NAM11	wt/wt	wt/wt	*NA*	wt/wt
NAM12	wt/wt	*NA*	wt/wt	wt/wt
NAM13	wt/wt	*NA*	*NA*	wt/wt
NAM14	wt/VAR	*NA*	*NA*	wt/VAR
NAM15	wt/wt	*NA*	*NA*	wt/wt
NAM16	wt/wt	*NA*	*NA*	wt/wt
NAM17	wt/wt	*NA*	*NA*	wt/wt
NAM18	wt/wt	*NA*	*NA*	wt/wt
NAM19	wt/wt	*NA*	*NA*	wt/wt
NAM20	wt/wt	*NA*	*NA*	wt/wt
NAM21	wt/wt	wt/wt	wt/wt	wt/wt
NAM22	wt/wt	*NA*	*NA*	wt/wt
NAM23	wt/wt	*NA*	*NA*	wt/wt
NAM24	wt/wt	*NA*	*NA*	wt/wt
NAM25	wt/wt	*NA*	*NA*	wt/wt
NAM26	wt/wt	*NA*	*NA*	wt/wt
NAM27	wt/wt	*NA*	*NA*	wt/wt
NAM28	wt/wt	*NA*	wt/wt	wt/wt
NAM29	wt/wt	*NA*	wt/wt	wt/wt
NAM30	wt/wt	*NA*	wt/wt	wt/wt
NAM31	wt/wt	*NA*	wt/wt	wt/wt
NAM32	wt/wt	wt/wt	*NA*	wt/wt
NAM33	wt/wt	*NA*	wt/wt	wt/wt
NAM34	wt/VAR	*NA*	wt/wt	wt/VAR
NAM35	wt/wt	*NA*	wt/wt	wt/wt
NAM36	wt/wt	*NA*	wt/wt	wt/wt
NAM37	wt/wt	*NA*	wt/wt	wt/wt
NAM38	wt/wt	*NA*	wt/wt	wt/wt
NAM39	wt/wt	*NA*	*NA*	wt/wt
NAM40	wt/wt	*NA*	*NA*	wt/wt
NAM41	wt/wt	*NA*	VAR/VAR	wt/wt
NAM42	wt/wt	*NA*	*NA*	wt/wt
NAM43	wt/wt	*NA*	*NA*	wt/wt
NAM44	wt/wt	*NA*	wt/wt	wt/wt

SAMPLE	rs1272632214	rs147390019	rs374594155	rs116855232
NAM45	wt/wt	wt/wt	*NA*	wt/wt
NAM46	wt/wt	*NA*	*NA*	wt/wt
NAM47	wt/wt	*NA*	*NA*	wt/wt
NAM48	wt/wt	*NA*	wt/wt	wt/wt
NAM49	wt/VAR	*NA*	wt/wt	wt/VAR
NAM50	wt/VAR	*NA*	*NA*	wt/VAR
NAM51	wt/VAR	*NA*	*NA*	wt/VAR
NAM52	wt/VAR	*NA*	wt/wt	wt/VAR
NAM53	wt/wt	*NA*	VAR/VAR	wt/wt
NAM54	wt/wt	*NA*	*NA*	wt/wt
NAM55	wt/wt	*NA*	wt/wt	wt/wt
NAM56	wt/VAR	*NA*	*NA*	wt/VAR
NAM57	wt/wt	wt/wt	wt/wt	wt/wt
NAM58	wt/VAR	*NA*	*NA*	wt/VAR
NAM59	wt/wt	*NA*	*NA*	wt/wt
NAM60	wt/wt	*NA*	VAR/VAR	wt/wt
NAM61	wt/wt	*NA*	*NA*	wt/wt
NAM62	wt/wt	*NA*	wt/VAR	wt/wt
NAM63	wt/wt	*NA*	wt/wt	wt/wt
NAM64	wt/VAR	*NA*	wt/wt	wt/VAR

NA = Not Available, missing data.

Anonymised dataset of the admixed Brazilian population

SAMPLE	rs1272632214	rs147390019	rs374594155	rs116855232
BAP1	wt/wt	wt/wt	wt/wt	wt/wt
BAP2	wt/wt	wt/wt	wt/VAR	wt/wt
BAP3	wt/wt	wt/wt	wt/wt	wt/wt
BAP4	wt/wt	wt/wt	wt/wt	wt/wt
BAP5	wt/VAR	wt/wt	wt/wt	wt/VAR
BAP6	wt/wt	wt/wt	wt/wt	wt/wt
BAP7	wt/wt	wt/wt	wt/wt	wt/wt
BAP8	wt/wt	wt/wt	VAR/VAR	wt/wt
BAP9	wt/wt	wt/wt	wt/wt	wt/wt
BAP10	wt/wt	wt/wt	wt/wt	wt/wt
BAP11	wt/wt	wt/wt	wt/wt	wt/wt
BAP12	wt/wt	wt/wt	wt/wt	wt/wt
BAP13	wt/wt	wt/wt	wt/wt	wt/wt
BAP14	wt/wt	wt/wt	wt/wt	wt/wt
BAP15	wt/wt	wt/wt	wt/wt	wt/wt
BAP16	wt/wt	wt/wt	wt/wt	wt/wt
BAP17	wt/VAR	wt/wt	wt/wt	wt/VAR
BAP18	wt/wt	wt/wt	wt/wt	wt/wt
BAP19	wt/wt	wt/wt	wt/wt	wt/wt
BAP20	wt/wt	wt/wt	wt/wt	wt/wt
BAP21	wt/wt	wt/wt	wt/wt	wt/wt

SAMPLE	rs1272632214	rs147390019	rs374594155	rs116855232
BAP22	wt/wt	wt/wt	VAR/VAR	wt/wt
BAP23	wt/wt	wt/wt	wt/wt	wt/wt
BAP24	wt/wt	wt/wt	wt/wt	wt/wt
BAP25	wt/wt	wt/wt	wt/wt	wt/wt
BAP26	wt/wt	wt/wt	wt/wt	wt/wt
BAP27	wt/wt	wt/wt	wt/wt	wt/wt
BAP28	wt/VAR	wt/wt	wt/wt	wt/VAR
BAP29	wt/wt	wt/wt	wt/wt	wt/wt
BAP30	wt/wt	wt/wt	wt/wt	wt/wt
BAP31	wt/wt	wt/wt	wt/VAR	wt/wt
BAP32	wt/wt	wt/wt	wt/wt	wt/wt
BAP33	wt/wt	wt/wt	wt/wt	wt/wt
BAP34	wt/wt	wt/wt	wt/wt	wt/wt
BAP35	wt/wt	wt/wt	wt/wt	wt/wt
BAP36	wt/wt	wt/wt	VAR/VAR	wt/wt
BAP37	wt/wt	wt/wt	wt/wt	wt/wt
BAP38	wt/wt	wt/wt	wt/wt	wt/wt
BAP39	wt/VAR	wt/wt	wt/wt	wt/VAR
BAP40	wt/wt	wt/wt	wt/wt	wt/wt
BAP41	wt/wt	wt/wt	wt/wt	wt/wt
BAP42	wt/wt	wt/wt	wt/wt	wt/wt
BAP43	wt/wt	wt/wt	wt/wt	wt/wt
BAP44	wt/wt	wt/wt	wt/wt	wt/wt
BAP45	wt/wt	wt/wt	wt/wt	wt/wt
BAP46	wt/wt	wt/wt	VAR/VAR	wt/wt
BAP47	wt/wt	wt/wt	wt/wt	wt/wt
BAP48	wt/wt	wt/wt	wt/wt	wt/wt
BAP49	wt/wt	wt/wt	wt/wt	wt/wt
BAP50	wt/wt	wt/wt	wt/wt	wt/wt
BAP51	wt/wt	wt/wt	wt/VAR	wt/wt
BAP52	wt/wt	wt/wt	wt/wt	wt/wt
BAP53	wt/wt	wt/wt	wt/wt	wt/wt
BAP54	wt/wt	VAR/VAR	wt/wt	wt/wt
BAP55	wt/wt	wt/wt	wt/wt	wt/wt
BAP56	wt/wt	wt/wt	wt/wt	wt/wt
BAP57	wt/wt	wt/wt	VAR/VAR	wt/wt
BAP58	wt/wt	wt/wt	wt/wt	wt/wt
BAP59	wt/wt	wt/wt	wt/wt	wt/wt
BAP60	wt/VAR	wt/wt	wt/wt	wt/VAR
BAP61	wt/wt	wt/wt	wt/wt	wt/wt
BAP62	wt/wt	wt/wt	wt/VAR	wt/wt
BAP63	wt/wt	wt/wt	wt/wt	wt/wt
BAP64	wt/wt	wt/wt	wt/wt	wt/wt
BAP65	wt/wt	wt/wt	wt/wt	wt/wt
BAP66	wt/wt	wt/wt	wt/wt	wt/wt

SAMPLE	rs1272632214	rs147390019	rs374594155	rs116855232
BAP67	wt/wt	wt/wt	wt/wt	wt/wt
BAP68	wt/wt	wt/wt	wt/wt	wt/wt
BAP69	wt/wt	wt/wt	wt/wt	wt/wt
BAP70	wt/wt	wt/wt	wt/wt	wt/wt
BAP71	wt/wt	wt/wt	VAR/VAR	wt/wt
BAP72	wt/wt	wt/wt	wt/wt	wt/wt
BAP73	wt/wt	wt/wt	wt/wt	wt/wt
BAP74	wt/wt	wt/wt	wt/wt	wt/wt
BAP75	wt/wt	wt/wt	wt/wt	wt/wt
BAP76	wt/VAR	wt/wt	wt/wt	wt/VAR
BAP77	wt/wt	wt/wt	wt/wt	wt/wt
BAP78	wt/wt	wt/wt	wt/wt	wt/wt
BAP79	wt/wt	wt/wt	wt/wt	wt/wt
BAP80	wt/wt	wt/wt	wt/wt	wt/wt
BAP81	wt/wt	wt/wt	VAR/VAR	wt/wt
BAP82	wt/wt	wt/wt	wt/wt	wt/wt
