# Supplementary material for: Identification of NUDT15 gene variants in Amazonian Amerindians and admixed individuals from northern Brazil
Source: PLoS One. 2020 Apr 15;15(4):e0231651. doi: 10.1371/journal.pone.0231651 (PMC7159207; doi:10.1371/journal.pone.0231651)
Supplement: Attachment — Submitted filename: Rebuttal Letter.pdf [file pone.0231651.s005.pdf]

Serviço Público
Federal Universidade Federal do Pará
Núcleo de Pesquisas em Oncologia

March 26th, 2020

Dear Reviewer #2,

A detailed item-by-item response follows. Our replies are marked in italic.

<u>Reviewer #2:</u> The authors have addressed all the concerns I had pointed in the previous submission. However, there are few issues I think that can still be improved.

1) Line 36. Non-admixed should be written instead of pure

Response: *The text has been modified accordingly.*

2) Line 123. genetic components should replace ethnic matrices

Response: *The text has been modified accordingly.*

3) Line 177. The R-packages used should be listed and cited. I.e. the R packages used to compute the FST. If no package was used for the FST computation and a self-made script was used, the FST formula should be written and a citation to the reference article added.

Response: *We did not use any R-package, in particular. The allele frequencies were obtained directly by gene. The Fisher's Exact Tests as well as the Bonferroni p-value correction was done by the RStudio version itself. And the FST calculation was realized by Arlequin v.3.5, we have added the referenced article, numbered as [21].*

4) Line 214. The p-values regarding the BAP population compared to the others should also be listed at Table 3. Moreover, the authors should apply a multi-test p-value correction. The p.adjust() function in the R package stats is one way to do it (although of course not the only one).

Response: *As requested, the BAP population was included in the pairwise comparisons of Table 3. Also, the p-values was adjusted by Bonferroni's method.*

5) Line 234. FST values should not be described as percentages

Response: *The manuscript has been modified accordingly.*

6) Line 237. BAP population must be included at table 4.
Response*: The manuscript has been modified accordingly.*

7) Line 261. Latin Americans with low percentages of Native American ancestry are not genetically similar to East Asians. In my opinion, this sentence is still not accurate. The authors can try a reformulation of the sentence to highlight that East Asians have the highest allele frequencies a part of Native Americans or directly replace exceeds 1% only in Asians and in populations genetically similar to them, that is, 262 the Latin and Amerindian populations by exceeds 1% only in Asians, Latin American and Amerindian populations.

Response*: We have reformulated the sentence accordingly.*

8) Line 216. The text should be rewritten regarding the new table 3.

Response*: We have rewritten about the new Table 3: Lines 227-235.*

9) Line 281. The authors should avoid the expression we believe

Response: *We have excluded the expression.*

10) Line 296. The results shown in this paragraph do not match with table 2.

Response*: We have not added the other five exonic mutations found in the ABraOM dataset because neither the Amerindians nor the admixed Brazilian population from Amazon (focus of our study) have these polymorphisms. Therefore, we have added the ABraOM website in the end of the paragraph, in case readers or the reviewer would like to check the additional information.*

11) Line 302. The results shown in this paragraph do not match with table 2.

Response*: The text has been corrected.*

12) Line 312. The authors should cite a paper where CLM, PUR, MXL and PEL populations are analyzed all together. Moreover, some of the articles cited have analyzed similar populations but not the 1000 genomes. The following articles

perform local ancestry analyses of CLM, PUR, MXL and PEL populations, while the last one, also includes local ancestry information for a South-Eastern Brazilian population.

https://journals.plos.org/plosgenetics/article?id=10.1371/journal.pgen.1004023

https://www.cell.com/ajhg/fulltext/S0002-9297(17)30107-6

https://www.nature.com/articles/s41598-019-50362-2

Response*: We appreciated and agreed with the suggestion. All the above-mentioned references have been included in our manuscript.*

13) Line 315 To be able to state "demonstrate" the authors should carry out a correlation test. Either the text should be adapted or the correlation performed

Response: *As requested, the text has been adapted.*

14) Supplementary table 1 is a presentation of the new data generated by the authors. Thus, the BAP population should be included (differentiated of the Native Americans), and therefore the second column can be renamed as population.

Response*: We added this suggestion and, therefore, have modified the supplementary table 1.*

15) Supplementary table 2 should also include BAP population

Response: *We included the BAP population in the supplementary table 2, as requested.*

16) A more coherent nomenclature can be used. I.e. a unified criteria to use the rs ID or the aminoacid subsitution. Moreover, the authors should reference the tables in the text when they discuss them explicitly.

Response*: We have modified the manuscript in accordance with this suggestion. The nomenclature used to the variants throughout the text is the rs ID.*

17) To conclude, the genetic information of the BAP population should also be available, together with the Native Americans.

Response: We included the raw data about the BAP population in the file named "Anonymised dataset".

Given the above, we hope that your suggestions have been addressed. Thank you for the attention.

Sincerely,

Ney Pereira Carneiro dos Santos, Ph.D. (Coordinator)

Hospital Universitário João de Barros Barreto – Núcleo de Pesquisa em Oncologia,

2º piso da Unidade de Alta Complexidade em Oncologia.

Av. Mundurucus, 4487, Guamá, 66073-005, Belém –PA.

Tel: +55 (91) 3201-6778
