# Supplementary material for: Identification of NUDT15 gene variants in Amazonian Amerindians and admixed individuals from northern Brazil
Source: PLoS One. 2020 Apr 15;15(4):e0231651. doi: 10.1371/journal.pone.0231651 (PMC7159207; doi:10.1371/journal.pone.0231651)
Supplement: Attachment — Submitted filename: Response to Reviewers.docx [file pone.0231651.s004.docx]

# Dear Reviewers

**Serviço Público**

**Federal Universidade Federal do Pará Núcleo de Pesquisas em Oncologia**

**January 24th, 2020**

A detailed item-by-item response to each of the Reviewers’ points follows. Our replies are marked in italic.

**Reviewer #1:** This is an interesting paper, which describes differences in frequencies of variants in NUDT15 gene among Amazonian Amerindians and admixed Brazilian samples, compared with 1000 Genome database. However, the main point of the paper, which is to relate NUDT15 variants with high frequency of toxic effects due to thiopurine treatment in northern Brazilian patients, were not achieve, since the authors only reports minimum allele frequencies, and did not perform any genetic association or similar analysis to compare patients with thiopurine toxic effects and controls, or with patients without thiopurine toxic effects. Below I describe additional Major and minor corrections:

1) Page 1, line 4: I would change the short title, since it to something like "Investigating NUDT15 gene variants in Amerindians and population from northern Brazil", since we cannot perform a "Whole-Exome" in one gene;

***Response:*** *We have changed the short title in accordance with this suggestion.*

2) Page 2, line 37: The authors should change this phrase, since only the coding region of NUDT15 gene was sequenced;

***Response:*** *We have changed the phrase to highlight that we did only the coding region of NUDT15 gene.*

3) Page 4, line 78: The authors should include a brief explanation about NUDT15 diplotypes;

***Response:*** *The text has been modified accordingly.*

4) Page 5, lines 98-101: The hypothesis seems confusing to me. Studies have already been shown that NUDT15 genotypes or haplotypes are related to toxic effects

from thiopurine-based treatments. However, here the hypothesis is exactly to see whether the link between NUDT15 genotypes and thiopurine toxic effects exists. I think what that the authors mean is whether the frequency of NUDT15 haplotypes is different in this specific population, which would explain the high frequency of severe toxicities. In addition, the authors cited "alternative NUDT15 genotypes", and what are these alternative genotypes? Therefore, I suggest that the authors rewrite this part, in order to better explain the hypothesis;

***Response:*** *We agreed that the manuscript's hypothesis was not very clear and therefore caused confusion about what we actually intended to accomplish. The objective of our paper was to describe the frequency of deleterious alleles of NUDT15 gene in a parental group forming of the actual population from northern Brazil (Native Americans) and in admixed northern population itself. We rewrote this part of the article and hope that the hypothesis has been cleared up.*

5) Page 5, lines 119-121: The authors should briefly explain what ancestry populations this admixed sample contains (Europeans, sub-Saharan Africans and Native Americans? or other additional ancestral one?). It could be achieved by global ancestry estimations (such as ADMIXTURE algorithm), in order to strength the ancestry population information;

***Response:*** *The main ancestries populations of the brazilian north population is the European, African and Native Americans. This data is well-stablished about the Brazilian population since it is a consequence of our colonization process. Also, this information has been confirmed by genetic analysis, using a dataset of 61 Ancestry Informative Markers.*

*Details about the methodology can be achieved in “Santos NP et al. Assessing individual interethnic admixture and population substructure using a 48-insertion- deletion (INSEL) ancestry-informative marker (AIM) panel. Hum Mutat. 2010;31(2):184-90.” and “Ramos et al. Neither self-reported ethnicity nor declared family origin are reliable indicators of genomic ancestry. Genetica. 2016; 144(3):259- 65.”)*

6) Page 6, line 137: In Bioinformatic analysis section, the authors should provide the database version of all database variant annotation used (ClinVar, LRT, Mutation

Taster, and others), regardless the variant annotation software used to achieved the database information (Annovar, VEP, VarStation, ViVa), since pathogenicity and clinical information could change depending on the database version used.

***Response****: The manuscript has been modified accordingly.*

7) Page 7, lines 152-153. The authors should report at least whether these variants were well covered by the exome. It could be included in one column in Table 1;

***Response****: We have included the information about the exome coverage in the first paragraph of the Results section.*

8) Table 1: Are the authors analyze other pathogenicity prediction algorithms rather than LRT and Mutation Taster? For example, SIFT, PolyPhen, CADD. If yes, they should be included in Table 1. If not, the authors should analyze the pathogenicity by these other algorithms and included the results in Table 1;

***Response****: All the pathogenicity prediction algorithms used are described in the subsection “Bioinformatic analysis” of the Methods section. We have not included them in the Table 1 because the only algorithms that showed pathogenicity predictions is the LRT and Mutation Taster.*

9) Page 8, lines 167-169: The difference in frequencies among the populations is very interesting; in this case, the authors should include a statistical test in order to guarantee that these differences are not by chance. If the frequency differences are not by chance, it also would be interesting to answer the question “Why are these frequencies are different in this specific population”. It could be due to some evolutionary processes, such as natural selection, genetic drift, or some problems in the data (too much related individuals, high inbreeding). I think the authors should explore these issues to better explain these high frequency (if it is not by chance);

***Response:*** *As the other reviewer also suggested, we performed a Fisher’s exact test to statistically evaluate the differences in populations frequencies observed. Also, we have explained in the Discussion section that we believe that these differences in frequencies may be due to genetic drift, a commonly genetic event observed in Amerindian populations.*

10) Page 9, line 179: The authors cited linkage disequilibrium estimation, but they did not describe how linkage disequilibrium was estimated in the methods. In addition, important variant information is lacking, for example, Hardy-Weinberg disequilibrium reports.

***Response****: We added a new subsection called “Statistical analysis” in the Methods section to better explain the tests and sofwtwares we have used in our data analyzes. The linkage disequilibrium was estimated using the Haploview software. Additionally, the Hardy-Weinberg disequilibrium reports has been described in the 198-200 lines.*

11) Page 11, lines 223-226: This phrase does not make sense, because the authors cited that no previous information about NUDT15 variation were report, and the next phrase cites one paper which one variant was investigated in Amerindians (ref 13);

***Response****: The text has been rewritten.*

12) Page 11, lines 230-231: The authors also should investigate the NUDT15 variation in the database provided by the Brazilian Initiative on Precision Medicine (BIPMed) (<https://bipmed.org/brave/index.html>);

***Response****: We agreed with the suggestion and data from the BIPMED database was included in our analyzes.*

13) Page 11, lines 239-240: The authors should clearly reference this information, since this information could be based on one paper only and could not reflect the southeastern Brazil samples;

***Response***: *We have supported this information using additional data about the subpopulation groups of the Latin population of the 1000 genomes database, i.e. Colombians, Mexican descendants, Peruvians and Puerto Ricans. The paragraph comprises the 308-317 lines.*

14) Page 12, lines 247-250: The hypothesis rise by the authors is that alternative NUDT15 genotypes could be involved in toxic effects observed in northern Brazilian patients exposed a thiopurine-based treatment. However, the authors did not perform any experiment to clearly link the presence of these NUDT15

haplotypes and levels of thiopurine: they just report the minor allele frequencies in the two groups (which seems that they were not exposed to thiopurine treatment). Therefore, these conclusions did not answer the objective of the paper, and I suggest that the authors design some experiment to answer this hypothesis, or change the hypothesis and the objectives of the paper.

***Response****: We clarified our hypothesis and hope that it now agrees with our results and conclusions;*

15) The authors should correct grammatical and typographical errors throughout the manuscript.

***Response***: *We sent the manuscript to be revised for a native English speaker professional translator, in order to correct typos and language errors across the text (a declaration has been provided and it is attached at the end of this document).*

**REVIEWER #2:** The authors address an interesting topic, that concerns the population based metabolism of thiopurine, which has important implications for the treatment of several diseases as Acute Lymphoblastic Leukemia or Inflammatory Bowel Disease. Admixed populations from northern Brazil have revealed much higher levels of toxicity than other world wide populations, according to the references cited by the authors.

The authors aim to identify the variants of the NUDT15 gene and assess the frequencies in two populations, a group of different samples belonging to several Native American populations from Northern Brazil and an admixed population from the same area. Moreover, the authors claim correlation of the Native American ancestry proportion in the admixed populations with the variants related to the high toxicity levels after the administration of theopurine based drugs.

I acknowledge the significance of the study. However, I want to highlight few points that the authors should address:

- First, the title of the manuscript is confusing. The title claims Whole exome sequencing of Amerindians and admixed individuals from northern Brazil. However, the study only analyses the NUDT15 gene. This is could be misleading. The authors should clarify if they have generated whole exome data. They also should explain why they only analyze this gene and not the rest of the exome. The title should not only be coherent with the text, but both should be consistent with the data provided.

***Commentary****: We have changed the title of the manuscript. Also, we have analyzed only the NUDT15 gene because we have a particular clinical condition in our region regarding the toxicities arising from thioupurine-treatments, that are dependent of the NUDT15 enzyme activity. So we performed a molecular epidemiology study in our population regarding the frequencies of deleterious alleles of this gene.*

Second. As the authors discuss, it is relevant to know if the pathogenic variants of the admixed populations are related to the Native American ancestry. However, there is a lack of evidence to support this hypothesis and the authors should perform the additional analyses needed for a solid discussion. Ideally, the authors should use the whole exome data to accurately assess how pathogenic variants are correlated with Native American genetic global and local ancestry.

Third, in the interest of personalized medicine, it would be interesting to see if some Native American populations have higher frequencies of the pathogenic alleles than others. Similarly, the frequencies in the American admixed populations can be correlated or not to the average proportions of the Native American ancestry, depending if the Native American genetic sources were different and had different frequencies of the pathogenic alleles.

Therefore, assuming the authors only have access to the data regarding the NUDT15 gene, the authors should address the following analyses.

1) An extra table for the allele frequencies independently for each of the 11 Native American populations when the population size is five or higher. This table should also analyze independently the four American admixed populations of the 1000 genomes and include the southeastern Brazilian admixed population. The authors should use this information to confirm and discuss more accurately what they say in line 227.

***Response****: We accepted this suggestion. The supplementary table 2 contains data requested. The discussion section has been modified accordingly the results achieved by the analyzes performed (lines 281-288).*

2) A table reporting the FST between populations regarding all variants of the region analyzed.

***Response****: We have accepted the suggestion and created the table 4, placed in the Results section.*

3) If the authors do not have access to the whole exome data and can not compute the average genomic proportions of ancestral components (Native American, European and sub-Saharan African) for the American admixed populations, they should at least report the values found in the literature for these populations (the amazonian Brazilian admixed population, the southeastern Brazilian admixed population and the four American admixed populations) and see if the frequency of the studied variants is correlated with the Native American ancestry as the authors claim in line 241.

***Response****: We have added the information requested. (lines 305-317).*

- Authors must also make minor but important changes: Introduction

1) Line 58: change ethnically by genetically

***Response:*** *The text has been modified accordingly.*

Methods

2) Line 114: add detailed information of the location of sampling of the admixed and Native American populations

***Response****: We have added the information requested (Supplementary table 1).*

3) Detail which regions are analyzed and in which chromosome they are found. Results

***Response****: The regions are the chromosome location are detailed in table 1.*

4) Sort tables 1 and 2 by position

***Response****: We have changed the tables in accordance with this suggestion.*

5) Paragraph starting in line 167 should be supported by a statistical test, like the Chi-squared test.

***Response****: We performed a Fisher’s exact test to support the differences in frequencies observed.*

6) Correct the sentence of line 206 where it is said that Native American populations are descendants of the analyzed East Asian populations. Native American might

be more closely related to East Asian populations than to other populations, but this does not mean they are their descendants.

***Response***: *We have corrected the sentence in accordance with this suggestion.*

Given the above, we hope that your suggestions have been adressed. Thank you for the attention.

Sincerely,

# Ney Pereira Carneiro dos Santos, Ph.D. (Coordinator)

Hospital Universitário João de Barros Barreto – Núcleo de Pesquisa em Oncologia,

2º piso da Unidade de Alta Complexidade em Oncologia. Av. Mundurucus, 4487, Guamá, 66073-005, Belém –PA.

Tel: +55 (91) 3201-6778

Avenida Desembargador João Bosco, 726

Atalaia 49.037-130 Aracaju – SE, Brazil

October 22nd, 2019

To whom it may concern,

I hereby confirm that I revised the text of the manuscript entitled “Identification of NUDT15 gene variants in Amazonian Amerindians and admixed individuals from northern Brazil.”, written by Juliana Rodrigues et al., and that the language is clear and free of grammatical errors. I am a native of Great Britain, with more than 25 years’ experience of research and teaching in Brazil, and more than 200 papers published in English language books and journals, as well as having translated or revised a number of hundred manuscripts for my colleagues.

Yours faithfully,

Stephen Francis Ferrari

E-ma[ils: ferrari@pq.cnpq.br,](mailto:ferrari@pq.cnpq.br) [stephenfferrari@gmail.com](mailto:stephenfferrari@gmail.com) ORCID: 0000-0003-0566-0000

Lattes curriculum[: http://buscatextual.cnpq.br/buscatextual/visualizacv.do?id=K4780092A2](http://buscatextual.cnpq.br/buscatextual/visualizacv.do?id=K4780092A2)
